# Harnessing Vaginal Probiotics for Enhanced Management of Uterine Disease and Reproductive Performance in Dairy Cows: A Conceptual Review

**DOI:** 10.3390/ani14071073

**Published:** 2024-04-01

**Authors:** Mounir Adnane, Ronan Whiston, Taurai Tasara, Ulrich Bleul, Aspinas Chapwanya

**Affiliations:** 1 Department of Biomedicine, Institute of Veterinary Sciences, University Ibn Khaldoun of Tiaret, Tiaret 14000, Algeria; 2 USDA, Faculty Exchange Program Fellow, University of Georgia, Athens, GA 30602, USA; 3 Department of Clinical Sciences, Ross University School of Veterinary Medicine, Basseterre 00265, Saint Kitts and Nevis; rwhiston@rossvet.edu.kn (R.W.); achapwanya@rossvet.edu.kn (A.C.); 4 Institute for Food Safety and Hygiene, Vetsuisse Faculty, University of Zurich, 8057 Zurich, Switzerland; taurai.tasara@uzh.ch; 5 Department of Farm Animals, Clinic of Reproductive Medicine, Vetsuisse Faculty, University of Zurich, 8057 Zurich, Switzerland; ubleul@vetclinics.uzh.ch

**Keywords:** probiotics, strains, uterine disease, cows, immune response, reproduction

## Abstract

**Simple Summary:**

Uterine health is crucial for cows to become pregnant and maintain farm profitability. However, when cows suffer from uterine diseases, it not only affects their fertility but also leads to increased antibiotic usage, impacting both animal health and farm economics. Probiotics, which are beneficial bacteria, offer a promising solution to improve cow health and reproductive success. Research suggests that certain probiotics can enhance cow fertility. Administering these probiotics directly into the cow’s vagina may strengthen uterine health, especially after giving birth. While initial findings are promising, further large-scale studies are needed to confirm their effectiveness. This paper underscores the importance of establishing clear guidelines for using probiotics in cow management, including selecting appropriate strains and administering them correctly. Moving forward, continued research is necessary to fully understand the benefits of probiotics in maintaining cow health and improving fertility.

**Abstract:**

Uterine disease in cattle impairs reproductive performance and profitability and increases antibiotic use and antimicrobial resistance. Thus, probiotics offer a promising alternative therapy. This review presents conceptual findings on the efficacy of probiotics in managing uterine diseases and fertility in cows. Probiotics containing *Lactobacillus* spp. and *Bifidobacterium* spp. individually or as composite formulations are known to improve fertility. Strategic intravaginal administration of these formulations would likely enhance uterine immunity, particularly during the postpartum period. While current findings on the benefits to uterine health are encouraging, there is still significant knowledge missing, including a lack of empirical information from large-scale field trials. This review underscores the need for evidence-based guidelines for probiotics, such as genomic selection of formulations, targeted delivery, or potential synergy with other interventions. Future research should address these gaps to maximize the potential of probiotics in managing uterine diseases and enhancing the reproductive health of dairy cattle.

## 1. Introduction

The periparturient transition from gestation to lactation involves crucial physiological adaptations to milk production and uterine involution [1]. The postpartum bovine uterus is usually contaminated with a range of culturable and unculturable microorganisms, but this is not always consistently associated with clinical diseases. Approximately 70% of all cows are susceptible to uterine disorders like metritis and endometritis that perturb uterine health, milk production, or reproductive performance [2,3,4,5,6,7]. Undisrupted uterine involution is pivotal for subsequent conception [8,9,10,11]. Endometritis is a common outcome of dysregulated uterine involution that impairs dairy profitability [12,13,14]. Currently, antibiotics are commonly used to treat sick animals. However, these pose challenges such as antimicrobial resistance and residues [15,16,17,18,19,20]. Therefore, probiotics could be a viable alternative therapy for mitigating uterine inflammations [21,22,23,24,25,26].

Because uterine involution is driven in part by the influx of bacteria into the reproductive tract at or after calving, understanding the uterine microbiota is critical to discovering therapies for uterine disease. It is known that the genital tract microbiome exerts a profound influence on endometrial health, homeostasis, and fertility [27,28]. While healthy cows’ vaginas harbor common microbial taxa, it is thought that a more diverse microbiome is associated with reproductive diseases [29]. The endometrial microbiome, which is more homogeneous compared to the vaginal microbiome, is fundamental for maternal recognition of pregnancy and maintenance of pregnancy [30,31,32,33].

Probiotic strains have been widely applied in the food and drug industries due to their potential health benefits. Probiotics have been suggested as therapies to improve growth, feed intake and efficiency, rumen fermentation, and immune and antioxidant capacities of cattle. Probiotics containing bacterial strains such as *Lactobacillus acidophilus (L. acidophilus)*, *L. casei*, *L. plantarum*, and *Bacillus subtilis* (*B. subtilis*) prevent uterine disease [34]. *L. acidophilus*, for instance, has anti-inflammatory and immune-boosting properties that enhance uterine immunity [35,36]. Significantly, the reproductive microbiota of bovines influences their uterine health and fertility. This review focuses on intravaginal probiotics in cows, exploring their utility in preventing uterine disease. By highlighting bovine-origin strains, we aim to provide insights into the benefits of the use of probiotics for uterine diseases in the context of preventing endometritis in cattle.

## 2. The Role of Uterine Microbiota in Bovine Reproductive Health

### 2.1. Normal Uterine Microbiota

Traditionally perceived as a sterile environment, the pregnant uterus has recently gained recognition as a host to various microorganisms [30,37,38]. Traditional culture-based microbiological approaches have limitations in that they cannot fully identify the whole genital microbiome [39,40]. The introduction of gene identification techniques has marked a paradigm shift in microbiome research, revealing a broader spectrum of bacterial species and providing a deeper understanding of this intricate microbial universe. The normal uterine microbiota of healthy postpartum cows is predominantly composed of the phyla *Bacteroidetes*, *Fusobacteria*, *Firmicutes*, *Proteobacteria*, and *Tenericutes* [29,41,42]. Influenced by factors such as age, parity, and the stage of the estrous cycle, its origin and composition are multifaceted [27]. These commensal bacteria employ various mechanisms, from competitive receptor occupation to biofilm generation, enhancing the cervicovaginal mucus barrier and thus thwarting pathogen proliferation [43,44]. Furthermore, they produce immune-active molecules like lactic acid, bolstering a harmonious vaginal microbiota while stymieing potential pathogen proliferation [45].

### 2.2. Dysbiosis and the Development of Uterine Inflammation

The delicate equilibrium of uterine microbiota is susceptible to disruption, leading to dysbiosis which increases the risk of uterine inflammation and subsequently, infertility [27,39,46]. In instances of dysbiosis, abundant pathogens may initiate an inflammatory response, causing damage to the endometrial epithelia [29,47]. Prominent pathogens causing metritis or endometritis in cattle include *Escherichia coli* (*E. coli*) and *Trueperella pyogenes* (*T. pyogenes*) [48,49]. Additionally, *A. pyogenes*, *F. necrophorum*, and *Prevotella* species are synergistic for uterine infections [50,51]. *F. necrophorum* secretes a potent leukotoxin that causes cell damage, while *Prevotella melaninogenicus* produces a compound that inhibits phagocyte function. Concurrently, *A. pyogenes* releases a growth-enhancing factor tailored for *F. necrophorum*, potentially enhancing its presence and impact within the uterine environment [10].

While fertility is intricately linked to uterine immunity, cytokine and chemokine secretion in response to bacteria can trigger uncontrolled inflammation. For example, granulosa cells recognize and respond to lipopolysaccharide (LPS), activating an inflammatory response within the follicular cells. This process affects steroidogenesis and oocyte development by inhibiting mitotic activity [52]. The symbiotic relationship between *E. coli*, *T. pyogenes*, and *F. necrophorum* involves the production of pyolysin by *T. pyogenes*, altering endometrial epithelial cell membranes, causing tissue damage, and disrupting mucosal integrity, leading to nidation issues [53]. Parturition presents a critical time for dysbiosis due to hormonal changes, bacterial influx, tissue damage, and subsequent uterine involutions [54]. The combination of pathogens and damage-associated molecular patterns (DAMPs) triggers an inflammation through the intracellular secretion of interleukin 1 (IL-1) by the endometrial epithelial cells [55]. 

Factors such as stress, malnutrition, metabolic disorders, or impaired immune responses can lead to dysbiosis, proliferation of pathogens, and uterine disease [56]. Stress releases cortisol, thereby suppressing the immune system [57]. Malnutrition and metabolic disorders, such as negative energy balance and ketosis, hinder immune cell functions and disrupt the genital tract microbiome [9,56,58]. Inflammation associated with dysbiosis is marked by elevated inflammatory cytokines IL-1β, IL-6, and TNF-α [59,60,61], tissue damage, and perturbed fertility [11]. Dysbiosis and proliferation of pathogenic bacteria decrease fertility by lowering conception rates and increasing gestational losses [11,62]. On the other hand, a healthy microbiome comprising diverse communities improve fertility [11]. Therefore, understanding the factors underlying dysbiosis is crucial for formulating effective preventative strategies for endometritis.

## 3. Overview of Probiotics

Probiotics have long been used for treating gastrointestinal and metabolic disorders in humans [63,64]. Recently, they have emerged as a viable option for uterine diseases in cattle. Additionally, their role as a promising delivery system for vaccines has been explored [65]. While probiotics have exhibited efficacy against diverse diseases, scant research has investigated their potential in treating reproductive diseases in animals, specifically in cattle [23,66]. Consequently, further exploration is essential to delineate optimal strains and conduct cost–benefit analyses for probiotic application in the management of genital health in dairy cattle.

### 3.1. Classification of Probiotics

**Figure 1:**
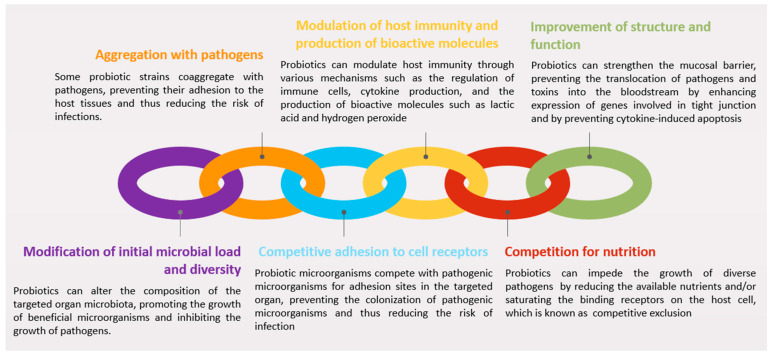
Schematic representation of common mechanisms of action required in probiotic bacteria for the management of uterine inflammation in cattle.

Based on general pharmacological concepts, probiotics can be categorized based on their origin, target host, or mechanism of action. The common classification system divides probiotics into three categories: (i) host-derived probiotics, which are strains of bacteria that naturally reside in the host’s gut or reproductive tract, (ii) food-derived probiotics, which are microorganisms added to food products, and (iii) pharmaceutical-derived probiotics, which are manufactured and administered as dietary supplements or drugs. Probiotics can also be classified into three classes according to the number of bacterial species: (i) monoprobiotics, which contain one strain of one bacterium, (ii) polyprobiotics, which contain different strains of one bacterial species, and (iii) combined probiotics, where different strains of different bacterial species could be combined in a single administration. Another classification system categorizes probiotics based on their mode of action (Figure 1). This system includes probiotics that work by (i) modifying the initial microbial load and diversity of the targeted organ, (ii) aggregating with pathogenic bacteria to encourage their ingestion by phagocytes, (iii) competitive adhesion to host epithelial receptors to reduce their access into the cell, (iv) producing immune-active molecules to reduce the growth of pathogens, (v) competing for available nutrients, which reduces the growth of invasive microbes, and (vi) modifying the structure and function of the organ (i.e., by affecting the hormonal profile) [67]. Irrespective of the classification used, the safety and efficacy of probiotics depend on factors like the strain, dose, and method of administration. Notably, bacterial metabolites or byproducts have potential as postbiotics for managing various disorders and enhancing animal growth and well-being. For instance, a study on postbiotic supplementation in newly weaned lambs demonstrated favorable outcomes, including improved weight gain, feed intake, and nutrient digestibility [68]. Consequently, rigorous selection and testing of probiotic strains are imperative before clinical application.

### 3.2. Mechanisms of Action

Due to the limited scope of extensive animal-centric research, we pivot our focus to studies conducted on humans. These investigations not only furnish robust evidence of efficacy but also serve as a foundation for contemplating their potential applicability and relevance in animal contexts. Adopting this approach allows for comprehensive exploration of therapeutic methodologies, enriching our understanding and facilitating applications across diverse organisms. The paucity of literature in this domain and the potential correlation between human and animal research motivate the elaboration of this conceptual review.

#### 3.2.1. Modification of Initial Microbial Load and Diversity

Drawing insights from human studies, various mechanisms and benefits of probiotics have been documented (Figure 1). Probiotics exhibit the capacity to reshape the microbiota of the targeted organ, fostering the proliferation of beneficial microorganisms while impeding the growth of pathogens. For example, in cattle, the oral use of a probiotic containing different combinations of *L. acidophilus* CRL2074, *L. fermentum* CRL2085, and *L. mucosae* CRL2069 resulted in alterations to the fecal microbiota [69]. Although beneficial outcomes have been observed from the application of probiotics within the bovine genital tract, the underlying mechanisms remain to be fully elucidated [23,66].

#### 3.2.2. Aggregation with Pathogens

Some probiotic strains possess the capability to aggregate pathogens, thereby impeding their adherence to host tissues and consequently reducing the risk of infections. In the absence of specific animal studies, insights from human research will be emphasized, serving as an indicator of the potential efficacy that may be extrapolated to animal models with the necessary adaptations. For instance, *L. rhamnosus* GG could potentially bind to the surface of *E. coli* in vitro, thereby potentially inhibiting its adhesion to intestinal epithelial cells [70]. Thus, the ability of probiotics to aggregate pathogens and inhibit their adhesion to host tissues, makes them a promising tool for preventing and treating infections caused by pathogenic microorganisms in different organs, including the genital tract.

#### 3.2.3. Competitive Adhesion to Cell Receptors

Probiotic microorganisms compete with pathogenic microorganisms for adhesion sites in the targeted organ, preventing the colonization of pathogenic microorganisms and thus reducing the risk of infection. In vitro studies have demonstrated that *Lactobacilli* can decrease IL-8 secretion by competing for cell-binding sites when transinfected with *H. pylori* [71]. Specific strains of *Lactobacilli* (*L. johnsonii* La1) and *Bifidobacteria* (*B. longum* SBT2928) share carbohydrate-binding specificities (i.e., Endo-H-treated yeast cell wall mannoprotein-carrying O-linked oligomannosides and gangliotri- and gangliotetra-osylceramides (asialo-GM1)) with enteropathogens such as *E. coli*, which are implicated in genital infections [72,73]. Furthermore, probiotic strains inhibit the attachment of pathogenic bacteria through steric hindrance at enterocyte pathogen receptors, as demonstrated by the inhibition of the internalization of enterohemorrhagic *E. coli* has been shown to be inhibited by *L. rhamnosus*, which is an adhering strain [74,75].

#### 3.2.4. Modulation of Host Immunity and Production of Bioactive Molecules

Probiotics exert immune-modulating effects through various mechanisms, influencing immune cells, cytokine production, and bioactive molecule secretion. Strains of *Lactobacilli* and *Bifidobacteria* impact immune function by regulating enterocytes, antigen-presenting cells, regulatory T cells, and effector T and B cells [76]. Bioactive molecules like short-chain fatty acids (SCFAs), hydrogen peroxide, and bacteriocins are produced by probiotics, exhibiting antimicrobial and anti-inflammatory properties [66]. *Lactobacilli*, which are known for carbohydrate metabolism to lactic acid, reduce organ pH, inhibiting the growth of pH-intolerant pathogens. SCFAs, particularly acetic and lactic acid, act as potent antimicrobial compounds against Gram-negative bacteria, reinforcing the inhibitory activity of probiotics against pathogens [77].

Probiotics stimulate the immune system, promoting cytokine production and the release of immune factors. The degradation of the bacterial component responsible for *Lactobacilli* adhesion results in antimicrobial peptides, enhancing host defense [78]. Bacteriocin-mediated killing commonly involves the destruction of target cells through pore formation or the inhibition of cell wall synthesis [79]. Bifidocin B is a bacteriocin produced by *B. bifidum* NCFB 1454 that exhibits activity against Gram-positive bacteria [80]. Furthermore, probiotics induce defensin secretion from epithelial cells, providing antimicrobial activity against various pathogens [81]. *Lactobacillus* synthesizes antifungal compounds like benzoic acid, methylhydantoin, and mevalonolactone [82,83]. Four antifungal substances produced by *L. plantarum* FST 1.7, including lactic acid, phenyllactic acid, and two cyclic dipeptides (cyclo(L-Leu-L-Pro) and cyclo(L-Phe-L-Pro)), have been characterized [84]. In addition to *Lactobacilli*, different strains of *Bifidobacterium* have been successfully used as probiotics for humans. A low-molecular-weight protein called BIF, which is produced by *B. longum* BL1928, exhibits antibacterial activity against Gram-negative bacteria by preventing the binding of *E. coli* to epithelial cells.

#### 3.2.5. Competition for Nutrients

Competitive exclusion, a natural phenomenon, occurs when microorganisms compete for resources, leading to the inhibition of less competitive organisms. In the bovine genital tract, the microbiota crucially maintains a healthy environment and prevents the growth of pathogenic bacteria [28]. This competitive microbial environment acts as a barrier, impeding the growth of harmful microorganisms. In vitro studies have demonstrated that *Lactobacilli* and *Bifidobacteria* hinder the growth of diverse pathogens, such as *E. coli*, *Salmonella*, *H. pylori*, *Listeria monocytogenes*, and *Rotaviruses*, possibly by reducing the available nutrients and/or saturating the binding receptors on the host cell [85]. 

#### 3.2.6. Improvement of Structure and Function

Probiotics can strengthen the epithelial barrier by preventing the translocation of pathogens or toxins into the bloodstream [86]. Upregulated expression of genes involved in tight junction signaling is thought to be the mechanism that reinforces the integrity of the epithelial barrier [87]. This then improves mucosal barrier function by reducing tissue permeability. *Lactobacilli* is known to upregulate genes encoding adherence junction proteins, E-catheridin and β-catenin [88]. Coincubation of enterocytes with *Lactobacilli* affects the phosphorylation of adherens junction proteins and the abundance of protein kinase C (PKC) isoforms (i.e., PKC δ) which would modulate epithelial barrier function [88]. *L. rhamnosus* CG is one of the most investigated bacteria in probiotics of the digestive system. This bacterium produces p40 and p75 proteins that prevent cytokine-induced cell apoptosis through the activation of anti-apoptotic protein kinase B (PKB/Akt) [89,90]. Thus, probiotics would effectively limit cytokine-induced epithelial damage, which is a hallmark of inflammatory conditions [91].

The mucosal surface of the genital tract is lined with mucus, forming a physical barrier that traps pathogens and prevents their attachment to the mucosa [92]. Mucus also contains immune-active molecules, including secretory immunoglobulins IgA, IgG, and IgM and α-defensins, β-defensins, lysozyme, lactoferrin, cathelicidin, calprotectin, and trappin-2/elafin [92,93]. Probiotics, particularly *Lactobacilli* strains, have been shown to influence the expression of genes related to mucin production by enterocytes [76,94,95,96]. This modulation suggests a potential role of probiotics in influencing the volume and composition of mucus, impacting the microbial diversity in the genital tract. Furthermore, certain components of probiotic bacteria, such as mucus-binding protein (MUB) and surface determinants like fimbriae-like appendages, play a crucial role in binding to the mucus layer. This binding promotes the colonization of beneficial bacteria in the mucosa, contributing to the health of the host [97,98,99]. While these mechanisms are predominantly drawn from studies on humans and related species, their potential implications for the genital tracts of cattle are considered, aligning with the reviewers’ suggestions.

### 3.3. Factors Affecting Probiotic Efficacy

**Figure 2:**
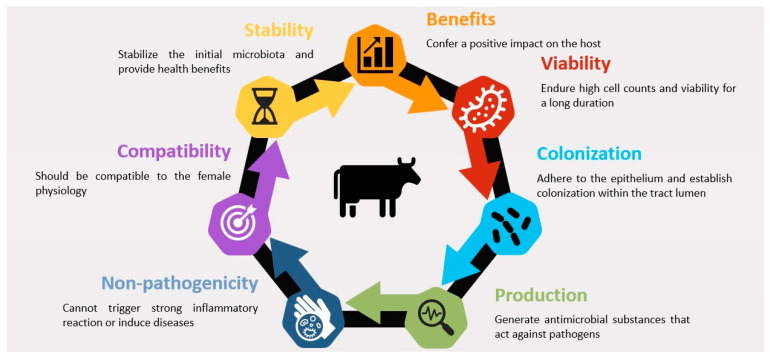
Factors affecting probiotic efficacy for the management of reproductive diseases in cows.

Probiotics intended for enhancing reproductive health in cows must fulfill specific criteria to ensure efficacy. These criteria include the ability to (i) positively impact the host, (ii) maintain high cell counts and viability over an extended period, (iii) adhere to the epithelium and establish colonization within the lumen of the genital tract, (iv) produce antimicrobial substances targeting pathogens, (v) stabilize the initial microbiota, providing health benefits, (vi) align with the female reproductive physiology, and (vii) exhibit non-pathogenic characteristics [64,67] (Figure 2). Crucially, effective probiotics should adhere firmly to the mucosa and mucus, facilitating the saturation of adhesion receptors and enabling aggregation with pathogens. This process modulates the immune response [75,100,101]. Common bacteria in probiotics, particularly lactic acid bacteria (LAB), possess adhesins, pili, and fimbriae surface components that facilitate interaction with epithelial cells and mucus in the gut and genital tract [99,100]. These surface components play a vital role in the initial attachment to the mucosa, which is a pivotal step for colonization and subsequent probiotic effects.

## 4. Probiotics for the Management of Uterine Disease in Cattle

The genital barrier, a critical defense system, encompasses the mucus layer, resident microbiome, resident immune cells, antimicrobial peptides, secretory IgA, and epithelial junction adhesion complex [102,103,104]. Any disruption in these protective barriers in the genital tract might favor the passage of pathogens and their toxins into the submucosa, leading to local inflammation and septicemia if the bacteria reach the blood stream [105].

### 4.1. Overview of Research on the Use of Probiotics for Uterine Health in Cattle

Direct-fed microbials (DFMs), which are oral probiotics for livestock [106], aim to prevent digestive diseases or enhance growth. Probiotics have been explored for managing uterine inflammation in cattle [19,23,66]. Emphasizing the effectiveness of intravaginal or intrauterine probiotic administration is crucial for delivering a sufficient bacterial load to the genital tract. Limited studies have investigated probiotics for uterine health in cattle [19,23,66], focusing on diverse strains and formulations and assessing reproductive outcomes, including pregnancy rates and conception time. For instance, intrauterine *L. buchneri* in cows correlated with higher pregnancy rates and reduced uterine infections, which were attributed to Lactobacillus inhibiting the LPS secretion of *E. coli*, thereby diminishing its mucosal adhesion [39,70,107]. 

### 4.2. Probiotic Strains and Formulations Used in Research

Commonly used probiotics for livestock, including cattle, encompass LAB, such as *L. bulgaricus*, *L. buchneri*, *L. acidophilus*, *L. casei*, *L. lactis*, *L. salivarius*, and *L. plantarum*, along with *Streptococcus thermophilus* (*S. thermophilus*), *Enterococcus faecium* (*E. faecium*), *E. faecalis*, and *Bifidobacterium* sp. [19,23,66,107,108]. *Aspergillus oryzae* and yeasts, such as *Saccharomyces cerevisiae*, are also employed [108]. Key probiotic strains studied for uterine health in cattle include various *Lactobacillus* and *Bifidobacterium* species. Different probiotic formulations have been tested, including intravaginal pessaries, intrauterine infusions, and oral supplements. Some of the probiotic strains and formulations that have been used in previous research are summarized in Table 1. LAB strains can be categorized as low adherent capacity in the endometrium, such as *L. sakei* and *L. reuteri*, or moderate-to-strong adherent capacity, such as *Pediococcus acidilactici* (*P. acidilactici*) and *L. rhamnosus*, according to their ability to form biofilms [109,110]. LAB can also be administered as probiotic mixtures where more than one species is used. A combination of *L. rhamnosus*, *P. acidilactici*, and *L. reuteri* in a 25:25:2 ratio had the highest potential to reduce inflammation on the endometrial epithelial cells and to decrease in vitro and ex vivo infections caused by *E. coli* [109,111]. These two studies confirmed that better modulation of inflammation is obtained when the bacterial species are combined, compared with the effect obtained by each strain individually. The probiotic mixture modulates the inflammatory reaction induced by *E. coli* by decreasing the gene expression and protein secretion of chemokine IL-8 and cytokines IL-1b and IL-6 [111] (Table 1). An in vivo study investigated the effects of intravaginal infusion of a probiotic mixture composed of *L. sakei* FUA3089, *P. acidilactici* FUA3138, and *P. acidilactici* FUA3140 [23]. This mixture was provided at a dose of 10^8^–10^9^ colony-forming units (CFUs) for each cow. The authors reported significant modulation of immune reactions and fewer uterine diseases after treatment. Moreover, studies exploring LAB strains like *Leuconostoc lactis*, *Ilyobacter polytropus*, *Enterococcus hirae*, and *Weissella confusa* from healthy cow vaginal microbiota showed potential probiotic properties [112]. Furthermore, the intravaginal administration of LAB was found to decrease the occurrence of metritis in dairy cows when compared to a control group [23]. Likewise, an equal combination of 10^10^–10^12^ CFUs containing *L. sakei* FUA 3089, *P. acidilactici* FUA 3140, and *P. acidilactici* FUA 3138 confirmed significant effects on improving productive and reproductive performances and reducing uterine inflammation [66] (Table 1). Therefore probiotics, including *L. acidophilus*, *L. rhamnosus*, and *E. faecium*, can be effective in managing reproductive diseases in cattle.

### 4.3. Obtaining Probiotic Strains and Formulations Used in Research

While various LAB strains are commercially available [109,111], potential drawbacks include their origin from niches like the gut, possibly lacking adaptation for the bovine genital tract. An alternative is isolating LAB strains from the uteri or vaginal mucus of healthy cows, ensuring suitability for reproductive health [107]. Another possible source is the vaginal mucus of healthy cows [102]. Preservation involves storing LAB strains at −80 °C in glycerol (15%) supplemented MRS broth. The preparation of microbiomes used for intravaginal administration was slightly different between the different experimental protocols described so far. For instance, the three LAB strains utilized in one of the described studies were individually cultivated. In that study, the *L. sakei* FUA 3089, *P. acidilactici* FUA 3140, and *P. acidilactici* FUA 3138 strains were separately cultivated in MRS broth cultures [66]. Subsequently, the bacterial cells were gathered via centrifugation at 5525× *g* for 20 min. The pellets of all three strains were then merged and resuspended in 10% skim milk. Aliquots of 250 µL of the probiotic mixture were freeze-dried at −70 °C using the freeze-dry system. A mixture of the three strains was stored in small vials at −20 °C. Prior to intravaginal administration, the freeze-dried LAB strain mixture was rehydrated in sterile physiological saline (0.9%) and administrated within two hours at a dose of 10^10^–10^12^ CFUs per cow [66]. A similar protocol was used by Deng et al. [23], where *L. sakei* FUA3089 and *P. acidilactici* FUA3138 and FUA3140 were mixed and stored in sterile skim milk with a cell count of 10^8^–10^9^ CFUs/dose. Probiotics were freeze-dried and frozen (−86 °C) in vials for storage. Before administration, the vials were similarly reconstituted in sterile physiological saline [23]. In a protocol described by Peter et al. [107], the *L. buchneri* DSM 32407 strain, isolated from the uterus of a healthy cow, was cultivated under aerobic conditions in MRS broth and then ultracentrifuged for 10 min and resuspended in MRS broth with glycerol, and small vials were created and stored at −80 °C. Before administration, the aliquots were thawed at room temperature and serial dilutions were cultivated on Rogosa SL agar under microaerophilic conditions at 37 °C and then stored at 4 °C. Before administration, and like the other two protocols, LAB (1.5–2 × 10^10^ CFUs) was reconstituted in isotonic saline solution (0.9%). These methods ensure the viability and efficacy of probiotic strains, offering a basis for further exploration in uterine health management in cattle.

### 4.4. Effects of Probiotics on Uterine Health and Fertility Outcomes

LAB treatment in multiparous cows enhances their conception rates compared to primiparous animals [66]. Notably, intravaginal probiotics not only improve fertility but also enhance milk production and feed intake [66,113]. This treatment modulates immune responses by reducing lipopolysaccharide-binding protein (LBP) and increasing IgA in vaginal mucus. Intravaginal administration of a mixture of LAB around calving reduces purulent discharges and plasma haptoglobin levels postpartum [66]. A mixture of *lactobacillus* and *Pediococcus* strains decreases uterine diseases [23]. Likewise, the intrauterine infusion of *L. buchneri* in cows with subclinical endometritis 24–30 days postpartum improved the first-service conception rate and reduced the median length of the calving–conception interval [107]. This treatment downregulates pro-inflammatory cytokines and chemokines, confirming the immunomodulatory role of LAB [43,107,114].

## 5. Factors Affecting the Efficacy of Probiotics for Uterine Inflammation

Probiotics can reduce the use of antibiotics for treating uterine diseases in cattle. However, many factors can affect the efficacy of probiotics in this context, including strain selection, dose, mode of delivery, and timing of administration.

### 5.1. Timing of Probiotic Administration

The timing of probiotic administration significantly influences its efficacy in managing uterine inflammation in cattle. Research underscores the critical importance of the peripartum period, a high-risk phase for metabolic disorders and uterine infections in cows [115,116,117]. Probiotics exhibit maximum efficacy when administered within the first two weeks after calving [23,66]. This timeframe aligns with the widened cervix, rendering the uterine environment more susceptible to bacterial infections. Intravaginal administration protocols vary, including pre-calving, post-calving, or both (Table 1). For instance, a mixture of LAB administered two weeks before calving and four weeks after calving demonstrated positive outcomes [66]. While in another study, different protocols of probiotic mixture infusion were compared; two doses of probiotics two weeks before calving and one dose one week after calving or two doses two weeks before calving only [23]. The authors reported similar results for the two protocols, which resulted in better management of uterine health compared to the controls. For the management of endometritis, LAB was administered directly into the uterine lumen at 24–30 days postpartum [23].

### 5.2. Route of Administration

The route of administration significantly influences the efficacy of probiotics in managing uterine inflammation in cattle. While oral administration is common due to its simplicity, research suggests that intravaginal and intrauterine routes may be more effective in delivering probiotics directly to the uterine site of infection [23,66]. Additionally, the intrauterine route can provide the most direct delivery of probiotics to the uterus. Intravaginal administration involves thorough vulva cleaning, disinfection, and aseptic probiotic solution delivery into the cranio-medial part of the vagina using a sterile insemination pipette and a 5 mL syringe [23,66]. Alternatively, intrauterine administration necessitates additional steps. A sterilized polytetrafluoroethylene tube (62 cm) is inserted through a metallic catheter passing through the cervix, ensuring aseptic administration to minimize contamination risks [107]. It is important to maintain strict aseptic techniques during the administration of the probiotic solution to prevent the introduction of pathogens into the uterus, which can cause further inflammation and infection.

### 5.3. Dosage of Probiotics

The administrated dose of probiotics is one of the most variable factors, as different dosages are proposed in different studies. In a study conducted on pregnant dairy cattle, each cow was intravaginally administered 10^10^–10^12^ CFUs of LABs [66] or 10^8^–10^9^ CFUs/dose per week. For intrauterine infusion, a dose of 1.5–2 × 10^10^ CFUs was proposed [107]. For better stability of the number of bacteria in each dose, the infusion should be administered within 8 h after preparation [107]. The thawed aliquots of the probiotic solution can be stably maintained for up to 48 h when stored at 4 °C. Similarly, the prepared solutions for intrauterine administration can be stored at room temperature for up to 8 h while still maintaining stable CFU levels [107]. It is important to note that the stability of CFUs in probiotic solutions can be influenced by various factors, such as the type of probiotic strain, the composition of the solution, and the storage conditions. Therefore, it is important to carefully monitor the stability of CFUs in the probiotic solution to ensure the efficacy of the treatment. Overall, the optimal dosage may vary depending on the specific probiotic strain, formulation used, and route and timing of administration. Furthermore, the determination of optimal probiotic doses and stability properties remains a subject of ongoing investigation. These areas require further optimization in future research endeavors. 

## 6. Current Gaps and Future Directions in Probiotics Research for Mitigating Uterine Disease in Cattle

The potential of probiotics for the management of uterine disease in cows is increasingly being recognized; however, due to the lack of precise, relevant, and conclusive findings, future research in this area is expected to focus on several key areas.

### 6.1. Identification of Optimal Probiotic Strains and Formulations

Research on probiotics for uterine disease in cattle must focus on pinpointing optimal strains and formulations. While *Lactobacilli* show promise, exploration beyond these strains is crucial, especially for those that are well adapted to the bovine uterine environment. Determining the ideal dose, duration, and delivery method is paramount. Employing a combination of in vitro and in vivo screening methods is a recommended approach. In vitro methods aid in identifying strains with desired functional properties, such as adhesion to epithelial cells, antimicrobial production, and immune modulation [109,111]. Subsequent in vivo studies should evaluate the efficacy of the identified strains in reducing uterine inflammation and enhancing reproductive outcomes in cattle [23,66,107,109]. Omics-guided approaches, including metagenomics, are instrumental in identifying optimal probiotic strains. For example, metagenomic analyses can differentiate microbial communities in healthy and diseased endometria in cattle [30,118]. By comparing these microbial communities, potential probiotic strains conducive to restoring a healthy endometrial microbiota can be identified. The focus of metagenomic approaches should extend beyond species identification to include predominant strains. Additionally, functional analyses such as meta-transcriptomics are vital for determining specific genes contributing to optimal reproductive system health and dysbiosis. Confirming the perturbations found in reproductive diseases is crucial to address potential technical biases, such as experiment design, sequencing technology limitations, or bioinformatic resource constraints (e.g., uncharacterized genes/proteins in commonly used databases). The recent release of MetaPhlAn4, with new microbial markers and the ability to profile a vast number of known and unknown microbial species, enhances the identification of optimal probiotic strains.

### 6.2. Development of Targeted Probiotic Delivery Methods

Developing targeted probiotic delivery methods is another critical area of research for effective probiotics for uterine inflammation in cattle. Although oral probiotic administration has demonstrated success in mitigating metabolic issues across species, its efficacy for reproductive problems may be limited [23,43,66,109]. Oral probiotics must overcome digestive processes to exert benefits in the uterus. One strategic approach involves employing intravaginal or intrauterine devices for direct probiotic delivery [66,107]. Intravaginal devices, such as sponges or pessaries, can be used to deliver probiotics to the vaginal environment, enabling colonization of the lower reproductive tract and preventing the ascent of pathogenic bacteria into the uterus. Intrauterine devices, such as controlled-release systems, can be used to deliver probiotics directly to the uterine environment, where they can reduce inflammation and improve reproductive outcomes. An innovative avenue for targeted probiotic delivery involves the use of probiotic-coated particles or nanoparticles that can be deposited in the uterus [119]. These particles can be designed to release probiotics in a controlled manner, allowing for sustained delivery over an extended period. This approach may improve the efficacy of probiotics by reducing the risk of premature clearance or degradation in the uterine environment.

### 6.3. Exploring Probiotic Interactions with Other Interventions

In advancing probiotics for mitigating uterine disease in cattle, an essential research area is probing their interactions with diverse interventions. This encompasses synergies with antibiotics, anti-inflammatory agents, and reproductive hormone therapies. A key inquiry revolves around the potential of probiotics to diminish the incidence of antibiotic-resistant bacterial infections. It is plausible that probiotics enhance antibiotic efficacy by facilitating deeper penetration into the uterine environment and mitigating the risk of biofilm formation. Also, probiotics may complement anti-inflammatory agents, such as nonsteroidal anti-inflammatory drugs (NSAIDs), in reducing uterine inflammation. By curbing the production of pro-inflammatory cytokines and fostering anti-inflammatory cytokines, probiotics may improve the anti-inflammatory effects of NSAIDs by reducing the production of pro-inflammatory cytokines and promoting anti-inflammatory cytokines, thereby improving reproductive performance. The influence of probiotics on reproductive hormone therapies remains a less-explored domain. Investigating whether probiotics enhance uterine receptivity and diminish the risk of inflammation-related implantation failure is crucial for comprehensive understanding. Additionally, exploring potential antagonistic effects is imperative. For instance, probiotics might compromise the efficacy of certain antibiotics by competing for binding sites or reducing absorption. Conversely, antibiotics may diminish the effectiveness of probiotics if the latter are susceptible to the antibiotics. Understanding these potential antagonistic effects is paramount for optimizing the combination of probiotics with other therapeutic modalities. 

## 7. Conclusions

Probiotic bacteria have the ability to combat endometritis infections caused by pathogens. In vitro and in vivo screening, combined with metagenomic analysis, hold promise for identifying novel probiotic strains that secrete metabolites exhibiting antimicrobial activity. Novel delivery methods are crucial for enhancing the efficacy of probiotics against uterine infections. While the intrauterine route offers direct probiotic delivery, it is invasive and less user-friendly. Investigating synergies with antibiotics, anti-inflammatory agents, and reproductive hormone therapies could potentially enhance reproductive outcomes while mitigating adverse effects. Probiotics may confer a distinct advantage, as they may be more adept at targeting the specific microbial population associated with endometritis. 

## Figures and Tables

**Table 1:** Summary of most-used probiotic strains for the management of uterine inflammation.

Probiotic Strains	Dosage	Study Type	Timing of Treatment	Route of Administration	Main Results	References
Mixture of 3 probiotic bacteria, *Lactobacillus sakei* FUA 3089, *Pediococcus acidilactici* FUA 3140, and *P. acidilactici* FUA 3138	10^10^–10^12^ CFUs	In vivo	2 weeks prepartum–4 weeks postpartum	Intravaginal	- Decreased purulent vaginal discharge - Decreased plasma haptoglobin - Increased pregnancy rate - Increased milk production	[66]
Mixture composed of *Lactobacillus sakei* FUA3089, *Pediococcus acidilactici* FUA3138, and *Pediococcus acidilactici* FUA3140	10^8^–10^9^ CFUs	In vivo	2 weeks prepartum and 1 week postpartum. or 2 weeks prepartum only	Intravaginal	- Lowered the incidence of metritis and total uterine infections - Lowered concentrations of systemic lbp - Increased vaginal mucus IgA	[23]
*Lactobacillus buchneri* DSM 32407	1.5–2 × 10^10^ CFUs	In vivo	24–30 days postpartum	Intrauterine	- Higher first-service conception rate - Shorter calving–conception interval - Lower expression of pro-inflammatory genes	[107]
*Lactobacillus rhamnosus, Pediococcus* *acidilactici*, *Lactobacillus reuteri*, and *Lactobacillus sakei* individually or in different combinations	Different doses	In vitro	Endometrial epithelial cells co-cultured with *E. coli*	-	- Reduced *E. coli* infection in vitro dependent on the dose and strain - Decreasing expression of IL8 and IL1β	[109]
Combination of *Lactobacillus rhamnosus, Pediococcus* *acidilactici*, and *Lactobacillus reuteri*	At a ratio of 25:25:2	In vitro—Ex vivo	Endometrial epithelial cells co-cultured with *E. coli*	-	- *E. coli* infection in vitro reduced by 89.77% - Decreasing IL-8, IL-1β, and IL-6	[111]

## Data Availability

Not applicable.
